# CDBProm: the Comprehensive Directory of Bacterial Promoters

**DOI:** 10.1093/nargab/lqae018

**Published:** 2024-02-21

**Authors:** Gustavo Sganzerla Martinez, Ernesto Perez-Rueda, Anuj Kumar, Mansi Dutt, Cinthia Rodríguez Maya, Leonardo Ledesma-Dominguez, Pedro Lenz Casa, Aditya Kumar, Scheila de Avila e Silva, David J Kelvin

**Affiliations:** Microbiology and Immunology, Dalhousie University, Halifax, Nova Scotia B3H 4H7, Canada; Pediatrics, Izaak Walton Killam (IWK) Health Center. Canadian Center for Vaccinology (CCfV), Halifax, Nova Scotia B3H 4H7, Canada; BioForge Canada Limited, Halifax, Nova Scotia B3N 3B9, Canada; Instituto de Investigaciones en Matemáticas Aplicadas y en Sistemas, Universidad Nacional Autonóma de México, Unidad Académica del Estado de Yucatán, Mérida 97302, Yucatán, Mexico; Microbiology and Immunology, Dalhousie University, Halifax, Nova Scotia B3H 4H7, Canada; Pediatrics, Izaak Walton Killam (IWK) Health Center. Canadian Center for Vaccinology (CCfV), Halifax, Nova Scotia B3H 4H7, Canada; BioForge Canada Limited, Halifax, Nova Scotia B3N 3B9, Canada; Microbiology and Immunology, Dalhousie University, Halifax, Nova Scotia B3H 4H7, Canada; Pediatrics, Izaak Walton Killam (IWK) Health Center. Canadian Center for Vaccinology (CCfV), Halifax, Nova Scotia B3H 4H7, Canada; BioForge Canada Limited, Halifax, Nova Scotia B3N 3B9, Canada; Facultad de Ciencias e Ingeniería, Universidad Nacional Autonoma de Mexico, Mexico City 04510, Mexico; Instituto de Investigaciones en Matematicas Aplicadas y en Sistemas, Universidad Nacional Autonoma de Mexico, Mexico City 04510, Mexico; Biotechnology Institute, Universidade de Caxias do Sul, Caxias do Sul, Rio Grande do Sul 95070-560, Brazil; Molecular Biology and Biotechnology, Tezpur University, Tezpur, Assam 784028, India; Biotechnology Institute, Universidade de Caxias do Sul, Caxias do Sul, Rio Grande do Sul 95070-560, Brazil; Microbiology and Immunology, Dalhousie University, Halifax, Nova Scotia B3H 4H7, Canada; Pediatrics, Izaak Walton Killam (IWK) Health Center. Canadian Center for Vaccinology (CCfV), Halifax, Nova Scotia B3H 4H7, Canada; BioForge Canada Limited, Halifax, Nova Scotia B3N 3B9, Canada

## Abstract

The decreasing cost of whole genome sequencing has produced high volumes of genomic information that require annotation. The experimental identification of promoter sequences, pivotal for regulating gene expression, is a laborious and cost-prohibitive task. To expedite this, we introduce the Comprehensive Directory of Bacterial Promoters (CDBProm), a directory of *in-silico* predicted bacterial promoter sequences. We first identified that an Extreme Gradient Boosting (XGBoost) algorithm would distinguish promoters from random downstream regions with an accuracy of 87%. To capture distinctive promoter signals, we generated a second XGBoost classifier trained on the instances misclassified in our first classifier. The predictor of CDBProm is then fed with over 55 million upstream regions from more than 6000 bacterial genomes. Upon finding potential promoter sequences in upstream regions, each promoter is mapped to the genomic data of the organism, linking the predicted promoter with its coding DNA sequence, and identifying the function of the gene regulated by the promoter. The collection of bacterial promoters available in CDBProm enables the quantitative analysis of a plethora of bacterial promoters. Our collection with over 24 million promoters is publicly available at https://aw.iimas.unam.mx/cdbprom/

## Graphical Abstract

**Graphical Abstract:**
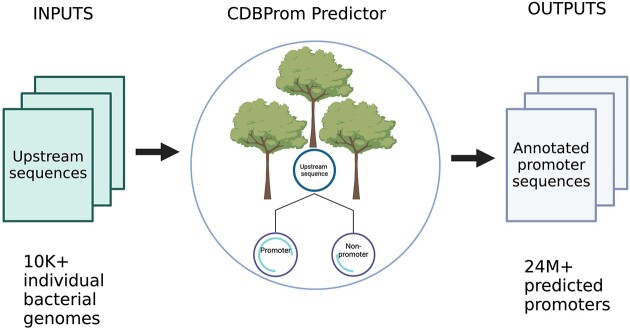

## Introduction

The transcription process is a crucial stage for bacterial cells to obtain necessary supplies and respond to environmental changes that may threaten their survival (1–3), including virulence factors that play a fundamental role in bacterial infection (4,5) . This process begins with the recruitment of the σ subunit of the DNA-dependent RNA polymerase (RNAP) enzyme and subsequent direction of the holoenzyme complex towards a DNA segment known as promoter. Promoters are conserved DNA sequences in which the RNAP σ subunits bind to. Examples of well-reported σ binding sites might be the positions –10, –35, extended –10, –12 and –24. However, it is worthwhile mentioning that consensus sequences of binding sites in promoters are degenerated (6), sometimes species specific (7), and patterns found in just a handful of sequences might not be extrapolated for the entire genome, which might lack functional annotation. These points make judging a sequence to be a promoter solely based on the presence or absence of the site a simplistic and reductionist approach that has been proven to be inaccurate (8,9). The physicochemical and structural features of the DNA molecule are also known to play a role in establishing ideal conditions for RNAP enzyme coupling and enabling the transcription process. These features include DNA duplex stability, DNA curvature, DNA enthalpy, and base-pair stacking (10), among others (11). Therefore, representing a regulator based on its structural features provides a more accurate representation of genetic information.

With the increasing availability and decreasing cost of high-throughput sequencing techniques, the amount of genomic information being deposited for analysis has surged (12). However, to handle this vast amount of data, automated processes that leverage artificial intelligence (AI) techniques are needed (13,14). AI has been successful in identifying patterns in molecular data and have facilitated the development of web tools (15), and databases (16,17) that offer valuable insights for clinicians and microbiologists. In particular, AI has greatly aided in the recognition of regulatory elements such as promoters (18,19) and other transcription factors (20,21), which play a crucial role in gene expression. By leveraging AI algorithms, we can effectively identify and analyze regulatory elements, enabling scientists to better understand the complex interactions within the genome.

In this study, our main goal is to generate a comprehensive database of bacterial promoter sequences publicly available for the scientific community. We aim to employ a two-sided machine learning approach that can learn the intrinsic factors around promoter sequences of a varied dataset as one of the factors of RNAP recognition of promoter sequences is the distinct levels of DNA Duplex Stability found in true promoter sequences. Moreover, external data will be used to ensure the generalization capacity of our classifier. Our database stands as a significant contribution to the field, representing one of the most extensive and all-inclusive resources of bacterial promoter sequences to have ever been made available.

## Materials and methods

### Train, test, validation and control datasets

We obtained 15 654 manually curated promoter sequences from previously published transcriptomic data to train and test our model. Each transcript was associated with its upstream region. We considered the position –60 to +1 relative to the TSS. This region has been reported as the core bacterial promoter, also known as the cis promoter element, necessary to initiate bacterial transcription (22,23). In Table 1, we include the name, accession number, number of promoter sequences obtained, and original reference for each organism of our train data.

**Table 1.** Description of the promoter sequences for training the model

Organism	Accession number (GenBank)	Number of promoters	Reference
*Anabaena* sp.	CP034058.1	2517	Unpublished
*Bacillus amyloliquefaciens* XH7	CP002927.1	1059	(24)
*Chlamydophila pneumoniae* CWL029	AE001363.1	404	(25)
*Corynebacterium glutamicum* ATCC 13 032	BA000036.3	575	Unpublished
*Helicobacter pylori* 26 695	CP003904.1	713	Unpublished
*Klebsiella pneumoniae* subsp. *Pneumoniae* MGH 78 578	CP000647.1	1329	(26)
*Mycobacterium tuberculosis* H37Rv	CP110619.1	1772	Unpublished
*Pseudomonas aeruginosa* UCBPP-PA14	CP034244.1	2054	Unpublished
*Salmonella enterica* subsp. *Enterica* serovar *Typhimurium* str. SL1344	CP133487.1	981	Unpublished
*Streptomyces coelicolor* A3	AL939104.1	2611	(27)
*Synechocystis* sp. PCC 6803 substr. GT-I	AP012276.1	1639	(28)

To have a balanced dataset that would enable the classification through machine learning approaches, we include an additional number of 15 654 non-promoter sequences. For obtaining negative sequences, we selected a same-sized sequence (i.e. 60 nucleotides sequence) from a random initial position (ranging from +1 to +500) in the downstream sequence where each promoter was located. The train/test data is available at 10.5281/zenodo.10581808.

To validate our model with unseen and independent sequences (i.e. not present either in the train or in the test of our model), we considered an additional set of 3861 experimentally verified promoter sequences from *Escherichia coli* K-12, available at the RegulonDB database (17). In a second validation step, we selected a reference benchmark dataset of bacterial promoters carved out by Zhang *et al.* (40) to test multiple classifiers. The bacteria present in the dataset comprises 283 promoters from *B. subtilis*, 98 promoters from *E. coli* (non σ70), and 1089 promoters from *E. coli* exclusively regulated by σ70. This dataset with 1470 promoters was also submitted to the classifier developed in this work to test its generalization capacity.

### Parametrization of genetic information

To enable machine learning methods to identify patterns in DNA information, a structural parametrization of promoter and non-promoter sequences was performed by assigning a DNA Duplex Stability (DDS) value to every dinucleotide in the sequences being converted. The theoretical basis behind this approach takes into account number of hydrogen bonds between the nucleotides, with adenine (A) and thymine (T) forming two hydrogen bonds and cytosine (C) and guanine (G) forming three hydrogen bonds. The difference in hydrogen bonding affects the chemical stability of the DNA molecule (29), and the conversion of promoter DNA sequences into DDS has been shown to effectively depict the AT rich elements that are commonly found in promoter sequences (10,30–32). This approach is also inclusive, as it accurately represents σ subunit binding sites in promoter sequences that lack apparent consensual motifs for the RNAP σ subunit binding to the promoter (6). We developed an in-house Python (version 3.9.7) script that converts *n* input promoter sequences into *n* output DDS coded promoter sequences. The script that performs this operation is publicly available at 10.5281/zenodo.10581808.

### Machine learning approaches

To classify between promoters and non-promoters, we assessed the performance of different methods, i.e. Random Forest, Support Vector Machines, Logistic Regression, Gradient Boosting, XGBoost, and K-Nearest Neighbors. All the algorithms were implemented using the scikit learn library (version 1.1.2) through the methods RandomForestClassifier, SVC, LogisticRegression, GradientBoostingClassifier, XGBClassifier, and KNeighborClassifier, respectively. To screen out the best algorithm, we selected the one that presented the best accuracy, precision, recall and F1 score.

We first determined the best model to classify between promoters and non-promoters. To develop a second instance of classification, we selected an additional model, trained with the misclassification of the first method (i.e. False Positives and False Negatives). After the algorithms were selected, each of them had their input parameters tested with the grid of parameters using the GridSearchCV method (scikit learn version 1.1.2). If the accuracy of the algorithm increased in comparison to the application of the default parameters, we selected the obtained best configuration of parameters. Moreover, we selected each model, obtained its test performance of the input data in a 10-fold cross validation step, averaged the result of each fold's accuracy, precision, recall, specificity, and Area Under the Curve (AUC) to depict the classification performance of each model. The Python code that implements the classifier of the Comprehensive Directory of Bacterial Promoters (CDBProm) is publicly available at 10.5281/zenodo.10581808. Moreover, the saved models that perform the two classification steps of CDBProm are also available at 10.5281/zenodo.10581808

To compare the prediction capacity of CDProm with other bacterial promoter predictors, we selected web tools that were available as of December 2023. The tools were selected according to their classification rationale, encompassing three steps of the natural gradation of classificatory tasks: (i) the tool BPROM (33) was selected as it employs traditional statistics (i.e. Linear Discriminant Analysis) as its form of classifying promoters; (ii) BacPP (14) and BDGP (34) were selected as they apply Machine Learning as a form of classification and (iii) Sapphire (35) was selected as it employs deep-learning to discriminate between promoters and non-promoters. We submitted a validation dataset comprising of 3861 *E. coli* promoters available at RegulonDB v.10.5 and measured the percentage of correctly classified promoters by each tool.

### Predicting novel promoter sequences

To data mine bacterial upstream regions and predict promoter sequences, we obtained data from 6812 unique bacteria deposited at the Regulatory Sequence Analysis Tool (RSAT) 2022 (16). We extracted the fasta files containing the sequences to be predicted as promoters by our method as well as a reference file that contains functional annotation for the upstream regions. Each upstream region deposited at RSAT spans from –400 to + 1 relative to the Coding DNA Sequence (CDS). We considered the span of –60 to +1, which is known to contain the main promoter for bacterial transcription to initiate (22,23) and matches the length of promoter regions found at RegulonDB (17). Each potential promoter sequence was treated individually. We first codified the sequences into DDS parameters and fed it to our classifier. For a potential promoter to be positively classified (i.e. a true promoter), it had to be assigned a label 1 by our two classifiers. The entirety of the predictions obtained with the application of the CDBProm classifier are available at https://aw.iimas.unam.mx/cdbprom/.

### Web server

The data of organisms were recorded into a relational MySQL database, in which two tables describing the genomic information of bacteria and archaea organisms are linked to the corresponding promoter predictions files. The master table contains the GCF as a key as well as the name of the organism, relating the kingdom, tax_id and species taxid. The web site was designed using the PHP language and the information can be accessed by genome name or by taxonomical classification, doing queries to it. Finally, raw files are included into the webpage to be downloaded.

## Results

### A two-sided classifier of bacterial promoter sequences

First, to screen the best classification algorithm for the promoter prediction task, we have submitted our train/test data, where 20% of the data was reserved for the test, into six different classification algorithms (Table 2), out of which XGBoost method was selected as it returned the best accuracy, precision, recall, and F1 scores.

**Table 2.** Comparison of distinct algorithms for classifying bacterial promoters

	Accuracy	Precision	Recall	F1 score
Random Forest	0.81	0.85	0.78	0.81
Support Vector Machines	0.74	0.74	0.75	0.75
Logistic Regression	0.74	0.74	0.75	0.75
Gradient Boosting	0.82	0.86	0.78	0.82
XGBoost	0.85	0.87	0.83	0.85
K-Nearest Neighbors	0.71	0.70	0.55	0.72

Next, to classify the train/test data with the XGBoost algorithm, we first iterated over a grid of hyperparameters, i.e. 50, 100 and 200 number of estimators; 0.01, 0.1, and 0.2 as learning rate; and 3, 5, and 7 as maximum depth. We report that a configuration of 200, 0.2 and 7 as number of estimators, learning rate, and maximum depth, respectively, resulted in the best model with an accuracy of 86%.

**Figure 1.**
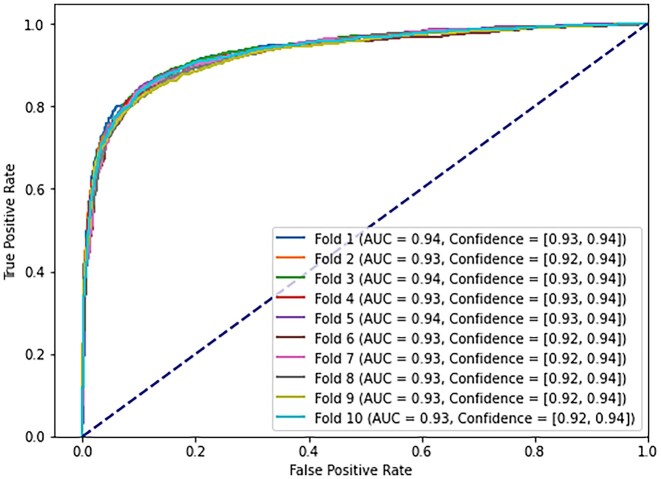
XGBoost classification method.

Moreover, to grant statistical feasibility of our XGBoost algorithm classifying different combinations of the training data, we have conducted the classification following a 10-fold cross validation step. We measured the testing accuracy, precision, recall, specificity (Table 3) and AUC (Figure 1), which returned, on average, scores of 0.87 ± 0.0, 0.88 ± 0.01, 0.85 ± 0.01, 0.88 ± 0.01 and 0.93 ± 0.01, respectively.

**Table 3.** Accuracy, precision, recall and specificity of each fold in the XGBoost classification

Fold	Accuracy	Precision	Recall	Specificity
1	0.86	0.88	0.85	0.88
2	0.86	0.87	0.84	0.88
3	0.87	0.87	0.86	0.87
4	0.87	0.87	0.85	0.89
5	0.87	0.89	0.85	0.89
6	0.86	0.89	0.85	0.88
7	0.87	0.88	0.84	0.89
8	0.86	0.89	0.84	0.88
9	0.86	0.87	0.83	0.89
10	0.87	0.88	0.85	0.89
Average	0.87	0.88	0.85	0.88
Standard deviation	0.0	0.01	0.01	0.01

**Figure 2.**
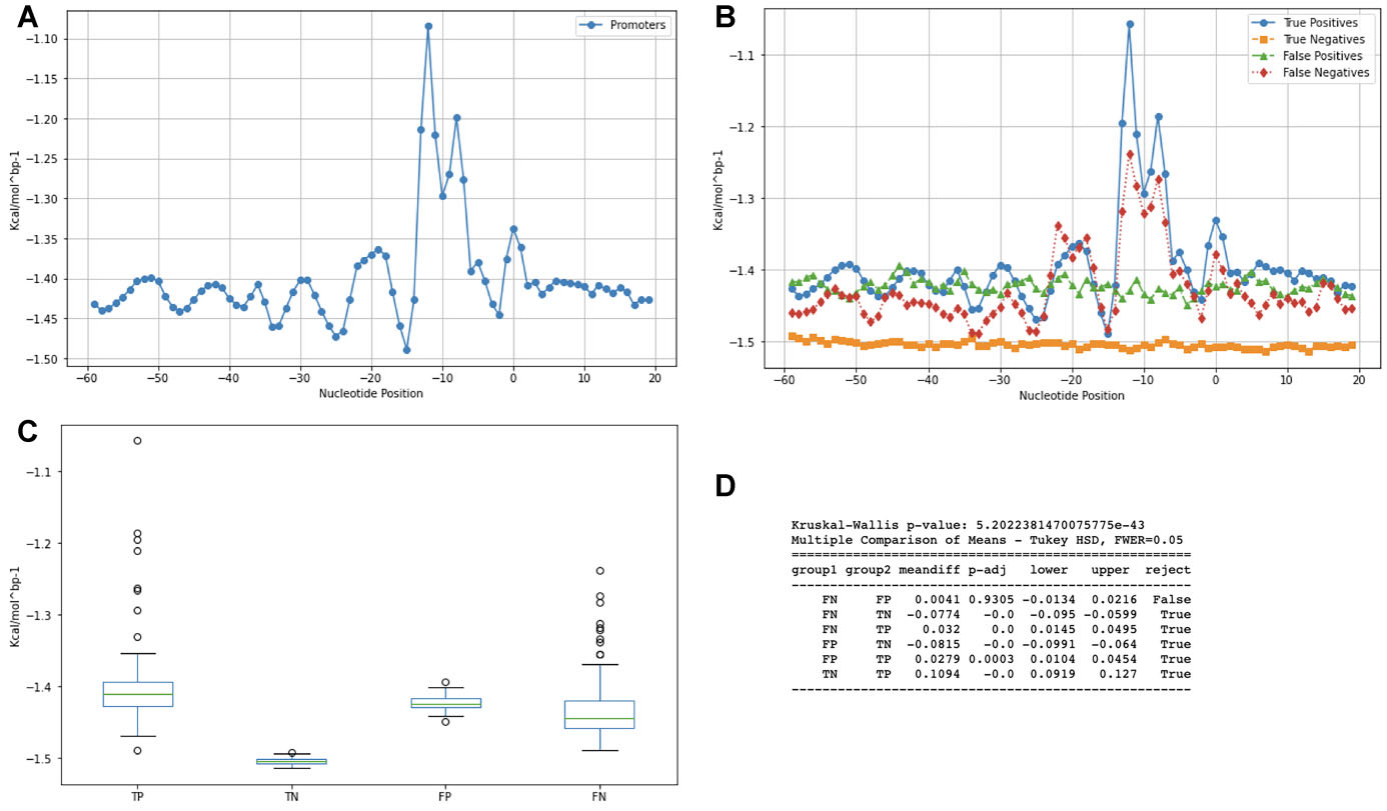
Mapping the decision pattern of the XGBoost model.

In addition, to enhance our classification capacity, we extracted the misclassified sequences from our XGBoost model. First, we determined an overall profile for 15 654 bacterial promoter sequences whose consensual motifs show where RNAP binds. As a result of our method, we observed that the -10 location was consistent with our DDS parametrization (Figure 2A).

In order to visualize the patterns of classification of our model, we mapped the DDS profile of True Positives (TPs, True Negatives (TNs), False Positives (FPs) and False Negatives (FNs) (Figure 2B). We report that the FNs (i.e. promoters classified as non-promoters, dotted red line) presented a lower peak in the positions –10 and –35. On the other hand, the FPs (i.e. non-promoters classified as promoters, dash-dotted green line) do not have the peaks at positions –10 and –35.

To compare the means of the TPs, TNs, FPs and FNs, we first applied a Shapiro-Wilk normality test, which returned a *P* value of 1.92e-17 denoting the data does not follow normal distribution. Next, we compared the means of TPs, TNs, FPs and FNs, group wise, with a Kruskal–Wallis test (Figure 2C), which returned a *P* value of 5.20e-43 (Figure 2D), indicating there are statistical differences, in general, between the groups. Finally, we compared the means of each group versus the others in a pairwise Tukey post hoc test and found there are no statistical differences only between FPs and FNs, whose *P* value is 0.93.

To achieve a second instance of classification, training with the error of the XGBoost classifier we selected all the 1810 FPs and combined with a random selection of 1810 FNs (out of 2406), constituting a balanced dataset in a 1:1 proportion. To determine the best classification algorithm for our second classification, we ran a suite composed of the algorithms Random Forest, Support Vector Machine, Logistic Regression, Gradient Boosting, XGBoost and K-Nearest Neighbors with default parameters and evaluated the accuracy, precision, recall and F1 score of the test data (i.e. 20%). We report that the algorithm XGBoost presented the best scores (Table 4); thus, it was chosen as the second step of our classifier.

**Table 4.** Comparison of distinct algorithms predicting the misclassification of the XGBoost algorithm

	Accuracy	Precision	Recall	F1 score
Random Forest	0.71	0.68	0.87	0.76
Support Vector Machines	0.65	0.64	0.78	0.70
Logistic Regression	0.66	0.65	0.79	0.71
Gradient Boosting	0.75	0.74	0.81	0.77
XGBoost	0.77	0.76	0.83	0.79
K-Nearest Neighbors	0.62	0.62	0.71	0.67

We then performed a hyperparameter tuning on the second XGBoost algorithm, iterating over 50, 100 and 200 number of estimators, 0.01. 0.1 and 0.2 as learning rate, and 3, 7 and 7 as maximum depth. The tuning on the hyperparameters did not result in a better accuracy score (i.e. 0.77) achieved with the default classification, thus, we preserved the default parameters.

**Figure 3.**
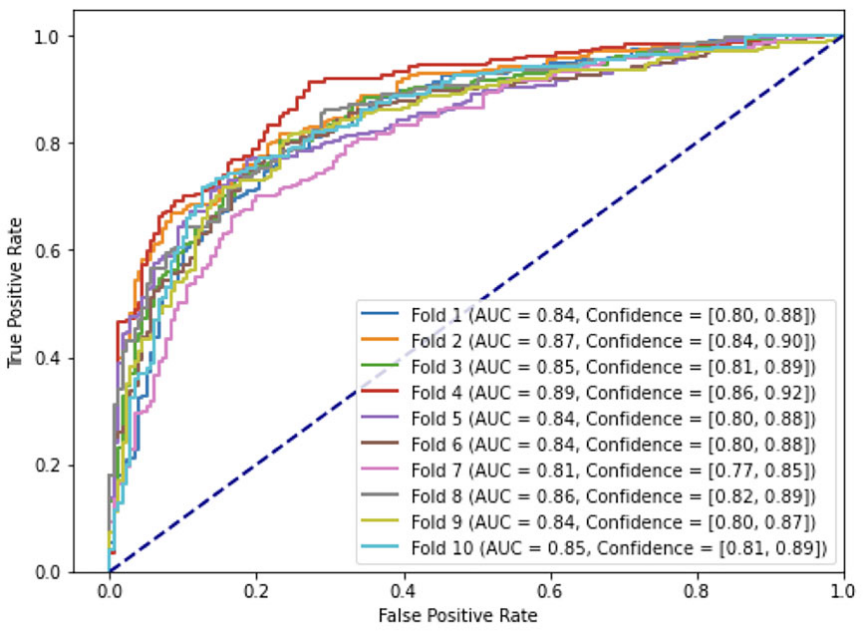
Second instance of the XGBoost classification trained and tested with the misclassified promoters from the first instance.

Next, we ran the second XGBoost with the train/test data separated into 10-folds for cross validation (Table 5). We report averaged accuracy, precision, recall, and specificity of 0.76 ± 0.02, 0.78 ± 0.02, 0.80 ± 0.03, 0.70 ± 0.03, respectively (Table 5) and AUC 0.84 ± 0.02 (Figure 3).

**Table 5.** Accuracy, precision, recall and specificity of each fold in the second XGBoost classification

Fold	Accuracy	Precision	Recall	Specificity
1	0.77	0.77	0.84	0.67
2	0.78	0.78	0.86	0.67
3	0.76	0.78	0.80	0.71
4	0.78	0.78	0.86	0.67
5	0.76	0.78	0.80	0.71
6	0.75	0.78	0.80	0.69
7	0.73	0.75	0.78	0.66
8	0.74	0.76	0.80	0.66
9	0.75	0.81	0.74	0.77
10	0.75	0.77	0.79	0.69
Average	0.76	0.78	0.81	0.69
Standard deviation	0.02	0.01	0.04	0.03

**Figure 4.**
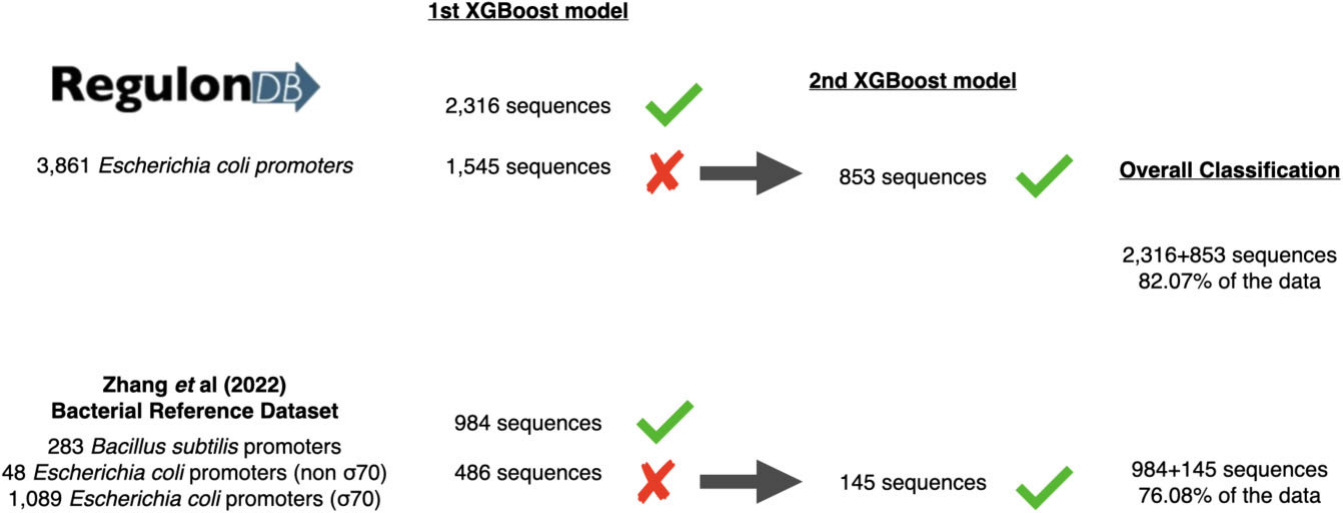
Validation of the CDBProm's XGBoost ensemble classification with external *Escherichia coli* promoter sequences.

Finally, in order to validate our two-sided model with unseen sequences, we obtained 3861 experimentally verified promoter sequences from the *E. coli* bacterium, available at RegulonDB v. 10.5 (17). We first submitted the 3861 promoters to our first instance of XGBoost, which correctly predicted 2316 sequences as promoters. Still, 1545 sequences were mislabelled, which were then submitted the second XGBoost instance, correctly identifying another 853 promoters. In total, 3169, 82% of the entire dataset was correctly classified as promoter sequences. Next, from the 1 470 promoters proposed as a reference dataset of bacterial promoter classification proposed by Zhang *et al.* (40), our first XGBoost captured 984 sequences while the second classified additional 145 sequences, totalling 76.08% of the entire dataset (Figure 4).

### Comparison of the prediction capacity of CDBProm's classifier with others

In order to compare the classification capacity of the algorithm of CDBProm with other available bacterial promoter predictors, we submitted the validation dataset (i.e. 3861 *E. coli* promoters from RegulonDB v. 10.5) to algorithms employing distinct classification techniques (Table 6). We report that a classification based on a Linear Discriminant Analysis (employed by BPROM) yielded the worst performance, correctly labelling only 818 out 3861 promoters. Next, there are three methods that employ machine learning as part of their classification, BDGP. BacPP, and our CDBProm. The three methods returned a classification performance of 57.9%, 74.3% and 82%, respectively. The three methods have distinct manners to codify the input information to submit to the classifier as BacPP employs an orthogonal classification and CDBProm codifies promoters as per the level of free-energy in-between dinucleotides; BDGP method of coding information to feed their Artificial Neural Network was not informed in the tool's documentation. Finally, the method of classification employed by Sapphire, i.e. a Deep Learning Convolutional Neural Network correctly classified 65.5% of the validation dataset.

**Table 6.** Comparison of distinct methods for classifying *E. coli* promoters

Tool	Predicted *E. coli* promoters	How they treat the input data	Method of classification
BPROM	818/3861 (21.1%)	Weight matrices of motifs	Linear discriminant analysis
BDGP	2236/3861 (57.9%)	Not informed	Machine learning
BacPP	2870/3861 (74.3%)	Orthogonal encoding	Machine learning
CDBProm	3543/3861 (82%)	Physico-chemical encoding	Machine learning
Sapphire	2529/3861 (65.5%)	Motif location with position-specific scoring matrices	Deep learning

### Annotating promoter sequences in 6000+ bacterial genomes

In total, we obtained 55 532 488 upstream sequences (26 171 424 from 6779 varied organisms and 29 361 064 from 5477 from diverse *E. coli*) from the RSAT Prokaryotes Database (16) . Each of these sequences was submitted to the classifier implemented in CDBProm. An upstream sequence as predicted as a promoter if it either the first or the second XGBoost predicted that sequence as a promoter. In addition, we also mapped each predicted promoter sequence to an additional file that contains annotation information about the upstream region (i.e. the gene regulated by the promoter). CDBProm's predictor identified 24 313 419 promoter sequences (12 274 077 from varied bacteria and 12 039 342 from *E. coli*). All the predictions are publicly available in CDBProm's webpage (https://aw.iimas.unam.mx/cdbprom/).

## Discussion

In our work, we delivered a large-scale directory of promoter sequences from varied bacteria as well as their regulatory annotation. In its current iteration, our database is composed of a collection of roughly five million predicted promoter sequences from 6779 organisms plus over five million promoter sequences predicted from distinct genomes of *E. coli*. We have also unified the novel bacterial promoter sequences of this study with a collection of archaeal promoters previously published (9). With the ever-growing availability of high-throughput sequencing methods, we should expect more strains of microorganisms to be sequenced, enabling the possibility of our database to be constantly updated. Moreover, the wide variety of bacterial promoters we delivered favors a qualitative/quantitative downstream analysis of their particularities, enabling the identification of RNAP σ subunit binding sites unique to specific microorganisms.

RegulonDB (17), PPD (36) and PromBase (37) are examples of promoter sequence databases that contain experimentally verified promoter sequences from different prokaryotic organisms. RegulonDB is a database that focuses on promoters from *E. coli* K-12 and provides information on the σ factor responsible for RNAP attachment to the promoter. The database currently has 4046 promoter sequences available for seven σ subunits. On the other hand, PPD contains over 129 419 experimentally verified promoters from 74 distinct prokaryotic organisms. Finally, PromBase counts with promoter sequences from 913 prokaryotic organism whose predictions were achieved by the PromPredict tool (30) These databases have been valuable resources for researchers studying bacterial genetics and regulation (38–40). Here, we argue that existing bacterial promoter databases might coexist and highly benefit from each other. In our work, a new database has been developed that contains over 5 million promoter sequences from over 6000 unique bacterial species. Our database is a valuable resource for researchers interested in exploring the diversity of bacterial promoters and their regulation across different organisms. Our database can benefit from existing databases such as RegulonDB, PPD and PromBase in a way that they can provide quality control measures and validation checks to ensure the consistency and accuracy of the deposited data, as both have experimentally verified sequences. On the other hand, existing databases can benefit from ours as it provides a substantially larger dataset to compare and validate their results.

In developing CDBProm, we opted to develop our own classifier, which granted us the full control of our pipeline. Despite the promoter prediction field owning a rich portfolio of diversified tools, we stress that our method provided a unique ensemble of two machine learning algorithms in which the second is trained with the error of the first. We argue that this provided a unique capacity for our tool to identify promoters with a less dominant signal as showed in Figure 2C. Out of the five tools we tested, CDBProm's algorithm presented the best classification performance in correctly labelling *E. coli* promoters.

Our study has certain limitations. First, the upstream sequences we have utilized for our analysis are sourced from a single database, RSAT (16). Although RSAT offers a wide range of prokaryotic organisms, we understand that having included input from diverse sources would have enhanced the ability of our tool to annotate an even larger database of potential promoters. Furthermore, we initially focused on developing CDBProm for bacteria only, even though RSAT contains upstream sequences from other biological kingdoms/domains such as archaea, protozoa, fungi, metazoan, and plants. Therefore, we consider this an opportunity to expand the scope of CDBProm in future iterations by incorporating promoter sequences from various biological kingdoms. Secondly, we did not provide a web tool for users to apply the characteristics we identified in promoters to predict their own potential promoter sequences. Despite the plethora of existing tools that can read input sequences and predict their probability of being a promoter (14,19,20), we are committed to further developing our own predictor in future iterations of our tool, as this would enable users to utilize our findings to predict potential promoters with greater accuracy. Finally, we also point out that our model was trained/tested with promoter sequences from one set of organisms and used to predict potential promoters in other bacteria. Despite the promoter region, specifically its binding sites, being prone to few insertions/deletions through evolution (41), we recognize that the transcription dynamics might be altered as per the distinct environments and metabolic needs of specific bacteria. Thus, in our directory, every predicted promoter has a label indicating that was a predicted promoter and there is no experimental validation for it.

Ultimately, we have been able to generate a comprehensive database of bacterial promoter sequences promptly available for the scientific community. The sequences we predicted have undergone a rigorous validation process to ensure their capacity well representing promoter activity.

## Data Availability

The entirety of datasets and codes used to warrant the conclusions of this study is publicly available at 10.5281/zenodo.10581808. Also, CDBProm database, containing the predicted promoter sequences obtained from this study is freely accessible at https://aw.iimas.unam.mx/cdbprom/.

## Notes

Present address: David J. Kelvin, Microbiology and Immunology, Dalhousie University, Halifax, Nova Scotia B3H 4H7, Canada.
